# Patterns and perceptions of vaping among adults living in social housing: a representative survey in Great Britain, 2023

**DOI:** 10.1186/s12889-024-20043-5

**Published:** 2024-09-20

**Authors:** Sarah E Jackson, Jamie Brown, Dan Lewer, Sharon Cox

**Affiliations:** 1 Department of Behavioural Science and Health, University College London, 1-19 Torrington Place, London, WC1E 7HB UK; 2 SPECTRUM Consortium, Edinburgh, UK; 3 Bradford Institute for Health Research, Bradford, UK; 4 Department of Epidemiology and Public Health, University College London, London, UK

**Keywords:** Social housing, E-cigarettes, Vaping, Vapes, Harm perceptions, Swap to stop, Inequalities

## Abstract

**Background:**

Vaping products are effective for helping people to stop smoking and may therefore offer a potential means to reduce high rates of smoking in socioeconomically disadvantaged groups. This study aimed to examine current patterns and perceptions of vaping among people living in social housing in Great Britain compared with those living in other housing types.

**Methods:**

Data were from the Smoking Toolkit Study; a nationally-representative survey conducted in 2023 (*n* = 23,245). Logistic regression tested cross-sectional associations between living in social (vs. other) housing and current vaping among adults; vaping frequency, device type, nicotine concentration, and source of purchase among current vapers; use of vaping products as a smoking cessation aid among past-year smokers who tried to quit; and harm perceptions of vaping products relative to cigarettes among current smokers.

**Results:**

Current vaping prevalence was twice as high among adults living in social housing (19.4%) compared with those in other housing types (10.4%; OR = 2.07, 95%CI = 1.84–2.33). This was partly explained by differences in sociodemographic characteristics and smoking status; after adjustment, the odds of being a current vaper were 33% higher (OR_adj_=1.33, 95%CI = 1.14–1.54). Among vapers, there were no notable differences by housing tenure in vaping frequency, main device type used, usual nicotine concentration, usual source of purchase, or use as a smoking cessation aid. However, current smokers living in social housing were more likely to think vaping is more harmful than cigarettes (31.6% vs. 21.8%; OR_adj_=1.61, 95%CI = 1.30–1.99).

**Conclusions:**

In Great Britain, adults who live in social housing are more likely to vape than those who live in other housing types, even after accounting for their younger age and higher smoking rates. However, misperceptions about the relative harms of vaping products and tobacco are common among smokers living in social housing. Interventions addressing these misperceptions could help encourage more people living in social housing to switch from smoking to vaping and reduce smoking-related health inequalities.

**Pre-registration:**

The study protocol and analysis plan were pre-registered on Open Science Framework (https://osf.io/n3mvs/).

**Supplementary Information:**

The online version contains supplementary material available at 10.1186/s12889-024-20043-5.

## Background

Smoking is a leading cause of disease, disability and premature death globally, killing up to two-thirds of those who do not quit [1–4]. One in every three premature deaths is attributable to socioeconomic inequalities, and the leading contributors are diseases associated with tobacco smoking [5–7]. Almost every indicator of socioeconomic disadvantage is associated with higher prevalence of smoking [8]. However, in England, housing tenure – specifically, living in social housing – is particularly strongly and independently linked with smoking [9, 10]. 

There is evidence from randomised controlled trials that vaping products (commonly referred to as e-cigarettes or vapes) can help people to stop smoking [11, 12]. Large, independent evidence reviews also consistently conclude that vaping is less harmful than smoking, but is not risk-free [13–16]. As such, encouraging people to switch from smoking to vaping is likely to reduce smoking-related health inequalities as long as interventions do not markedly increase uptake of vaping among people who would not have otherwise smoked. Understanding current patterns and perceptions of vaping among adults living in social housing can inform the design and delivery of interventions to encourage this population to switch from smoking to vaping.

In Great Britain, social housing is offered at reduced rental rates on a secure, long-term basis to people who cannot afford to rent or buy a home on the open market. Priority is given to those with the most urgent housing needs. Social housing is regulated and funded by the government and owned and managed by local authorities (local councils comprising publicly elected councillors) or housing associations (independent, not-for-profit organisations). A nationally-representative survey in England in 2015-17 found that smoking rates were more than twice as high among adults living in social housing (34%) than those living in other housing types (e.g., home owners or private renters; 15%) [10]. Notably, despite being similarly motivated to quit, smokers living in social housing were only around half as likely to be successful when they tried [10]. The pattern of results was similar when those living in social housing were compared with each other housing type separately (i.e., owned outright, bought on a mortgage, privately rented, or other) [10]. Similar findings were also observed when the analysis was updated including data up to 2020, with no indication that this disparity was improving over time [17]. This evidence highlighted the need for targeted action to address these inequalities [18]. 

In April 2023, the UK government announced a national ‘Swap To Stop’ campaign, which aims to offer one million people a free vaping starter kit with behavioural support to help people stop smoking and is likely to be targeted to less advantaged populations [19]. The scheme was inspired by a pilot programme in Salford, Greater Manchester in 2018, which offered adult smokers living in social housing a voucher that could be exchanged for a free e-cigarette from a local pharmacy or stop smoking service [18]. There were increased numbers of smokers attending local stop smoking services and successfully quitting smoking, particularly in the most deprived quintile [18]. The national Swap To Stop initiative will run in collaboration with local authorities: each will design the scheme to suit their needs, including deciding which populations to prioritise [19]. Social housing residents are likely to be an important target, given their high smoking rates [10, 17] and the success of Salford’s pilot in this group [18]. It will be important to understand the current vaping context among adults living in social housing in order to promote uptake and tailor support. Specifically, there is a need for up-to-date information on who in this population is vaping, the types of products they are using, where they are typically purchasing them, the extent to which they are being used to support smoking cessation, and how smokers perceive the harms of vaping relative to smoking.

Using data from a nationally representative survey of adults in Great Britain in 2023, this study aimed to examine current patterns and perceptions of vaping among people living in social housing. Specifically, we aimed to address the following research questions:

1. Among all adults, how does the prevalence of vaping among those living in social housing compare with other housing tenures?
2. Among current vapers, is living in social housing associated with vaping frequency, main type of device used, usual nicotine concentration, or source of purchase?
3. Among past-year smokers who have tried to stop smoking, is living in social housing associated with the likelihood of using vaping products as a smoking cessation aid?
4. Among current smokers, is living in social housing associated with perceptions of the relative harms of vaping products and cigarettes?

## Methods

### Pre-registration

The study protocol and analysis plan were pre-registered on Open Science Framework (https://osf.io/n3mvs/). In addition to our pre-registered analyses, we also calculated unadjusted and adjusted odds ratios for current vaping by housing tenure within subgroups of age, gender, occupational social grade, and country within Great Britain to provide a more detailed picture of differences between groups living in social housing compared with other housing types. Following peer review, we added an unplanned analysis in which we separated out private renters from the ‘other housing’ comparator (given evidence showing many within this group are under financial stress [20]) and analysed housing tenure as a 3-level variable (social renters, private renters, home owners).

### Design

Data were drawn from the Smoking Toolkit Study, a monthly cross-sectional survey that recruits a representative sample of adults (≥ 16 years) in Great Britain [21, 22]. The study uses a hybrid of random probability and simple quota sampling to select a new sample of approximately 2,450 adults each month. Data are collected via computer-assisted telephone interviews. Comparisons with other national surveys and sales data indicate that key variables such as sociodemographic characteristics, smoking prevalence, and cigarette consumption are nationally representative [21, 23]. 

For the present study, we used data from respondents to the survey in the period from January 2023 (the first wave since February 2020 in which housing tenure was assessed) to October 2023 (the most recent data available at the time of analysis). Respondents in England were only asked about vaping frequency, nicotine concentration, and source of purchase in certain waves in 2023 (January, April, June, July, and October) due to availability of competitive research funding, so analyses of these outcomes were restricted to data collected in these waves.

### Measures

#### Housing type

Housing type was categorised as ‘social housing’ (homes belonging to a housing association or rented from local authority) vs. ‘other housing’ (homes bought on a mortgage, owned outright, rented from private landlord, or other; reference category). We also reported the prevalence of current vaping within each separate housing type. In an unplanned analysis, we categorised housing type as ‘social housing’, ‘private rented’, and ‘home owner’ (bought on a mortgage or owned outright; reference category).

#### Sociodemographic characteristics

Age, gender, occupational social grade, and country were recorded. Age was analysed as a categorical variable (16–24/25–34/35–44/45–54/55–64/≥65 years), to account for non-linear associations with vaping and smoking. Gender was self-reported as man or woman. In more recent waves, participants have also had the option to describe their gender in another way; due to low numbers those who identified in another way were excluded from analyses stratifying by or adjusting for gender. Occupational social grade was assessed using the National Readership Survey classification [24] and categorised for analysis as ABC1 (includes managerial, professional, and upper supervisory occupations) or C2DE (includes manual routine, semi-routine, lower supervisory, and long-term unemployed); we also provided descriptive data on the composition of the sample using a more granular categorisation (AB/C1/C2/D/E). Country was categorised as England, Wales, or Scotland.

#### Smoking status

Participants were asked which of the following best applies to them:

- ‘I smoke cigarettes (including hand-rolled) every day’.
- ‘I smoke cigarettes (including hand-rolled), but not every day’.
- ‘I do not smoke cigarettes at all, but I do smoke tobacco of some kind (e.g. pipe, cigar or shisha)’.
- ‘I have stopped smoking completely in the last year’.
- ‘I stopped smoking completely more than a year ago’.
- ‘I have never been a smoker (i.e. smoked for a year or more)’.

Those who responded *a*,* b*, or *c* were considered current smokers, those who responded *d* or *e* ex-smokers, and those who responded *f* never-smokers (never regularly smoked). For the analysis of use of vaping products in a quit attempt, those who responded *a*,* b*,* c*, or *d* were considered past-year smokers.

#### Vaping status

Vaping status was assessed within several questions asking about use of a range of nicotine products. Current smokers were asked ‘Do you regularly use any of the following in situations when you are not allowed to smoke?’ and those who reported cutting down ‘Which, if any, of the following are you currently using to help you cut down the amount you smoke?’; past-year smokers (including current smokers) were asked ‘Can I check, are you using any of the following either to help you stop smoking, to help you cut down or for any other reason at all?’; and non-smokers were asked ‘Can I check, are you using any of the following?’. Those who reported using an e-cigarette in response to any of these questions were considered current vapers.

#### Vaping frequency

Current vapers were asked: ’How many times per day on average do you use your nicotine replacement product or products?’ People who reported vaping at least once a day were considered to be vaping daily (versus non-daily). Those who responded ‘don’t know’ were excluded.

### Main device type

Current vapers were asked: ‘Which of the following do you mainly use…?’ Response options were:

- *Disposable* – ‘A disposable e-cigarette or vaping device (non-rechargeable)’.
- *Refillable* – ‘An e-cigarette or vaping device with a tank that you refill with liquids (rechargeable)’ or ‘A modular system that you refill with liquids (you use your own combination of separate devices: batteries, atomizers, etc.)’.
- *Pod* – ‘An e-cigarette or vaping device that uses replaceable pre-filled cartridges (rechargeable)’.

We dummy coded these categories for analysis (i.e., disposable vs. all other; refillable vs. all other; pod vs. all other).

### Usual nicotine concentration

Current vapers were asked: ‘Does the electronic cigarette or vaping device you mainly use contain nicotine?’ with response options yes, no, and don’t know. Those who responded yes to this question were asked: ‘What strength is the e-liquid that you mainly use in your electronic cigarette or vaping device?’ We reported descriptive data on the following response categories:

- No nicotine.
- 6 mg/ml (~ 0.6%) or less.
- 7 mg/ml (~ 0.7%) to 11 mg/ml (~ 1.1%).
- 12 mg/ml (~ 1.2%) to 19 mg/ml (~ 1.9%).
- 20 mg/ml (~ 2.0%) or more.
- Don’t know.

For regression models, we excluded those who responded ‘don’t know’ and analysed other response options as an ordinal variable.

### Usual source of purchase

Current vapers were asked: ‘From where do you usually buy your disposable e-cigarette or vaping device, pre-filled cartridges, e-liquids or electronic cigarette?’

Response options included the following types of retailer:

- *Vape shop* – ‘Specialist vape / electronic cigarette retailer – not online’.
- *Online vape retailer* – ‘Specialist vape / electronic cigarette retailer – online’.
- *Other online retailer* – ‘Other online retailer’.
- *Newsagent* – ‘Newsagent/off licence/corner shop’.
- *Petrol station* – ‘Petrol garage shop’.
- *Supermarket* – ‘Supermarket’.
- *Friend* – ‘Buy them cheap from friends’.
- *Other* – ‘Other’ or ‘Don’t know’.

For our analyses, we classified the responses into four categories:

- Vape shop (response *a*).
- Supermarket/convenience store (*d-f*).
- Online (*b* and *c*).
- Other (*g-h*).

We dummy coded these categories for analysis (i.e., vape shop vs. all other; supermarket/convenience store vs. all other; online vs. all other; other vs. all other).

### Use of vaping products as a smoking cessation aid

Past-year smokers were asked: ‘How many serious attempts to stop smoking have you made in the last 12 months? By serious attempt I mean you decided that you would try to make sure you never smoked again. Please include any attempt that you are currently making and please include any successful attempt made within the last year’. Those who reported having made at least one attempt to quit were asked: ‘Which, if any, of the following did you try to help you stop smoking during the most recent serious quit attempt?’. Participants were asked to indicate all that apply. Those who responded ‘electronic cigarette’ were considered to have used vaping products as a smoking cessation aid.

### Harm perceptions of vaping products

Current smokers (only) were asked: ‘Compared to regular cigarettes, do you think electronic cigarettes are more, less, or equally harmful to health?’ Response options were ‘more harmful’, ‘less harmful’, ‘equally harmful’, or ‘don’t know’. We dummy coded these response options for analysis, with less harmful (vs. all other responses) as our primary outcome for this variable, consistent with current evidence that e-cigarettes are less harmful than cigarettes [13], and equally harmful (vs. all other), more harmful (vs. all other), and don’t know (vs. all other) as secondary outcomes.

### Statistical analysis

Data were analysed in R v.4.2.1. We excluded participants with missing data on housing tenure. Missing cases on other variables were excluded on a per-analysis basis (see table footnotes for details). The Smoking Toolkit Study uses raking to weight the sample to match the population of Great Britain in terms of key demographics. These key demographics are determined each month using data from the UK Census, the Office for National Statistics mid-year estimates, and the National Readership Survey [21]. The following analyses used weighted data.

We reported the prevalence of current vaping by housing tenure (social housing/other [ref]), among all adults and stratified by age, gender, occupational social grade, country, and smoking status. We also reported the prevalence of current vaping among all adults living in other housing, stratified by housing type (bought on a mortgage/owned outright/rented from private landlord/other).

Among all adults, we used binary logistic regression to test associations of housing tenure (social housing/other [ref] and social housing/private rented/home owner [ref]) with current vaping, with and without adjustment for sociodemographic characteristics and smoking status. We repeated the models comparing social housing with other housing tenures stratified by age, gender, occupational social grade, country, and smoking status to explore differences between subgroups.

Among current vapers, we used regression models to test associations of housing tenure (social housing/other [ref] and social housing/private rented/home owner [ref]) with vaping frequency (binary logistic regression), main device type (dummy coded as one-versus-rest; binary logistic regression), usual nicotine concentration (ordinal logistic regression), and source of purchase (dummy coded as one-versus-rest; binary logistic regression), with and without adjustment for sociodemographic characteristics and smoking status.

Among past-year smokers who tried to stop smoking, we used binary logistic regression to test associations of housing tenure (social housing/other [ref] and social housing/private rented/home owner [ref]) with use of vaping products in the most recent quit attempt, with and without adjustment for sociodemographic characteristics.

Among current smokers, we used binary logistic regression to test associations of housing tenure (social housing/other [ref] and social housing/private rented/home owner [ref]) with harm perceptions of vaping products compared with cigarettes (dummy coded as one-versus-rest), with and without adjustment for sociodemographic characteristics and vaping status. In an unplanned analysis, we reran the analysis comparing social housing with other housing tenures stratified by vaping status, to explore differences between exclusive smokers and those who also used vaping products (‘dual users’).

## Results

A total of 23,977 (unweighted) adults aged ≥ 16 years in Great Britain were surveyed between January and October 2023. We excluded 732 (3.1%) with missing data on housing tenure. This left a final sample of 23,245 participants, of whom 3,161 (13.6%) lived in social housing and 20,084 (86.4%) lived in other housing types (9,514 [40.9%] owned outright, 6,864 [29.5%] bought on a mortgage, 3,221 [13.9%] privately rented, and 485 [2.1%] other).

Table 1 presents weighted sample characteristics stratified by housing tenure. On average, participants living in social housing were younger (45.0 vs. 48.9 years) than those living in other housing types, more were women (57.3% vs. 49.5%) and from less advantaged occupational social grades (C2DE; 71.3% vs. 39.3%), fewer lived in Wales (3.6% vs. 5.2%), and more were current smokers (28.3% vs. 14.3%).

**Table 1:** Sociodemographic characteristics and smoking status by housing tenure (*n* = 23,245)

	Housing tenure	
	Social housing	Other housing
Unweighted *N*	3,161	20,084
Age (years)		
Mean [SD]	45.0 [18.5]	48.9 [18.5]
16–24	17.7 [16.1–19.5]	11.5 [10.9–12.1]
25–34	19.3 [17.7–21.0]	16.2 [15.5–16.8]
35–44	15.5 [14.1–17.0]	16.0 [15.4–16.6]
45–54	14.7 [13.4–16.1]	16.6 [16.0–17.1]
55–64	14.3 [13.1–15.7]	16.0 [15.5–16.6]
≥65	18.4 [17.1–19.8]	23.8 [23.2–24.5]
Gender		
Men	41.6 [39.6–43.6]	49.8 [49.0–50.6]
Women	57.3 [55.3–59.3]	49.5 [48.8–50.3]
Other	1.1 [0.8–1.5]	0.7 [0.6–0.8]
Occupational social grade		
ABC1 (more advantaged)	28.7 [27.1–30.3]	60.7 [59.9–61.5]
AB	7.0 [6.1–8.0]	30.0 [29.3–30.8]
C1	21.7 [20.3–23.1]	30.7 [30.0–31.4]
C2DE (less advantaged)	71.3 [69.7–72.9]	39.3 [38.5–40.1]
C2	20.9 [19.2–22.6]	20.3 [19.6–21.0]
D	25.7 [23.6–27.7]	12.5 [11.8–13.1]
E	24.7 [23.1–26.3]	6.5 [6.1–6.8]
Country		
England	87.3 [86.3–88.2]	86.1 [85.7–86.5]
Wales	3.6 [3.2–4.2]	5.2 [4.9–5.4]
Scotland	9.0 [8.3–9.9]	8.7 [8.4–9.0]
Smoking status		
Never smoker	44.6 [42.6–46.7]	59.6 [58.9–60.4]
Ex-smoker	27.0 [25.3–28.8]	26.0 [25.3–26.7]
Current smoker	28.3 [26.5–30.2]	14.3 [13.8–14.9]
Exclusive smoker*	69.5 [65.9–73.1]	67.8 [65.7–69.9]
Dual user of tobacco and e-cigarettes*	30.5 [26.9–34.1]	32.2 [30.1–34.3]

Data are presented as column percentages with 95% confidence intervals unless otherwise specified. There were some missing data (age *n* = 10, gender *n* = 104, smoking status *n* = 120); valid percentages are shown. * Proportion of current smokers.

### Vaping prevalence

**Fig. 1:**
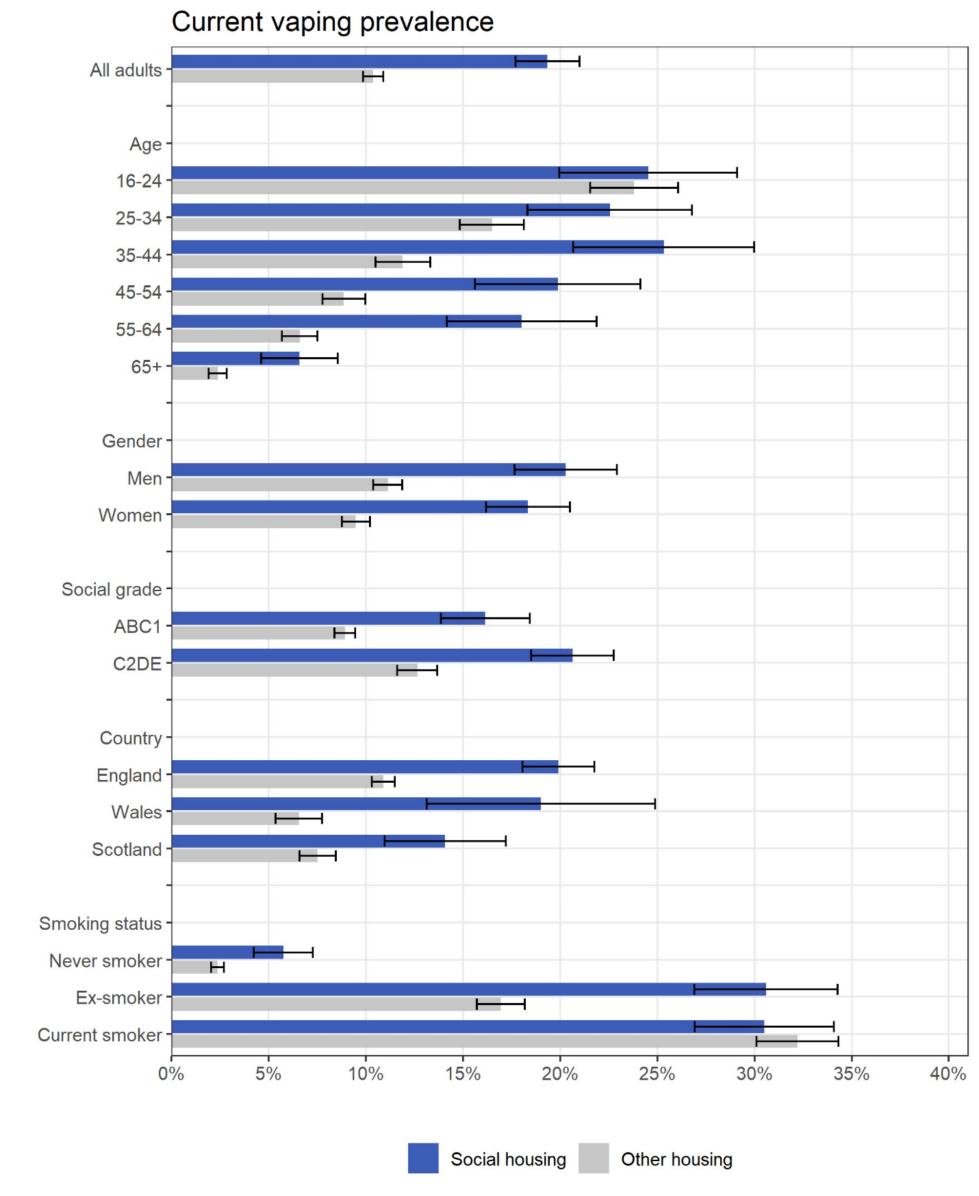
Prevalence of current vaping by housing tenure (*n* = 23,245). Data are provided in tabular form in Table S1. Unadjusted and adjusted odds ratios are provided in Table 2

Vaping prevalence was twice as high among adults living in social housing compared with those in other housing types (19.4% vs. 10.4%; Fig. 1, Table S1). When we analysed other housing types separately, vaping prevalence was also higher among those living in private rented accommodation (18.0% [95%CI 16.5–19.6%]) compared with those who owned their own home – either bought on a mortgage (10.7% [9.8–11.6%]) or owned outright (6.3% [5.7–6.9%]) – or who lived in other accommodation (12.5% [9.0–16.1%]).

The extent of differences in vaping prevalence by housing tenure varied by age and smoking status (Fig. 1). The disparity was more pronounced among middle-aged and older adults (≥ 35 years), with similar rates in the youngest group (16–24 years). Rates of vaping were higher among never smokers and ex-smokers living in social housing vs. other housing types, but were similar across housing tenures among current smokers. Differences in vaping prevalence by housing tenure were similar by gender, occupational social grade, and country (Fig. 1).

After adjustment for age, gender, occupational social grade, country, and smoking status, the odds of vaping were 33% (95%CI 14–54%) higher among adults living in social housing compared with other housing types (OR_adj_=1.33, 95%CI 1.14–1.54; Table 2). Compared with home owners, the odds of vaping were 40% (95%CI 20-64%) higher among adults living in social housing (OR_adj_=1.40, 95%CI 1.20–1.64). Those living in private rented accommodation also appeared to have slightly higher odds of vaping compared with home owners, but this difference was uncertain (OR_adj_=1.16, 95%CI 1.00-1.35).

When we repeated the analysis stratified by smoking status, living in social housing (vs. other housing) was associated with higher odds of vaping for adults who had never regularly smoked (OR_adj_=1.62, 95%CI 1.13–2.31) and for those who were ex-smokers (OR_adj_=1.70, 95%CI 1.35–2.13), but the odds of vaping were similar by housing tenure for current smokers (OR_adj_=0.93, 95%CI 0.75–1.16; Table 2).

**Table 2:** Unadjusted and adjusted associations of housing tenure with current vaping, overall and among population subgroups (*n* = 23,245)

	Current vaping (social housing vs. other housing types [ref])	
	Unadjusted OR [95% CI]	Adjusted OR [95% CI]^1^
All adults	2.07 [1.84–2.33]	1.33 [1.14–1.54]
Age (years)		
16–24	1.04 [0.79–1.37]	0.86 [0.61–1.21]
25–34	1.48 [1.13–1.93]	1.02 [0.74–1.41]
35–44	2.51 [1.90–3.32]	1.67 [1.19–2.36]
45–54	2.55 [1.89–3.44]	1.44 [1.01–2.03]
55–64	3.11 [2.31–4.20]	2.01 [1.43–2.83]
≥65	2.89 [1.99–4.21]	1.70 [1.10–2.63]
Gender		
Men	2.03 [1.70–2.43]	1.50 [1.20–1.86]
Women	2.15 [1.82–2.53]	1.19 [0.97–1.47]
Occupational social grade		
ABC1 (more advantaged)	1.97 [1.64–2.35]	1.32 [1.04–1.66]
C2DE (less advantaged)	1.80 [1.53–2.11]	1.34 [1.11–1.61]
Country		
England	2.03 [1.78–2.32]	1.36 [1.15–1.60]
Wales	3.35 [2.18–5.13]	1.75 [0.98–3.11]
Scotland	2.02 [1.51–2.70]	0.89 [0.62–1.27]
Smoking status		
Never smokers	2.53 [1.85–3.47]	1.62 [1.13–2.31]
Ex-smokers	2.16 [1.78–2.62]	1.70 [1.35–2.13]
Current smokers	0.92 [0.76–1.12]	0.93 [0.75–1.16]

CI, confidence interval. OR, odds ratio.

^1^ Adjusted for age, gender, occupational social grade, country, and smoking status.

There were some missing data (age *n* = 10, gender *n* = 272, smoking status *n* = 120), so samples sizes for analyses by these variables and for the adjusted analyses were slightly smaller.

### Vaping characteristics

**Fig. 2:**
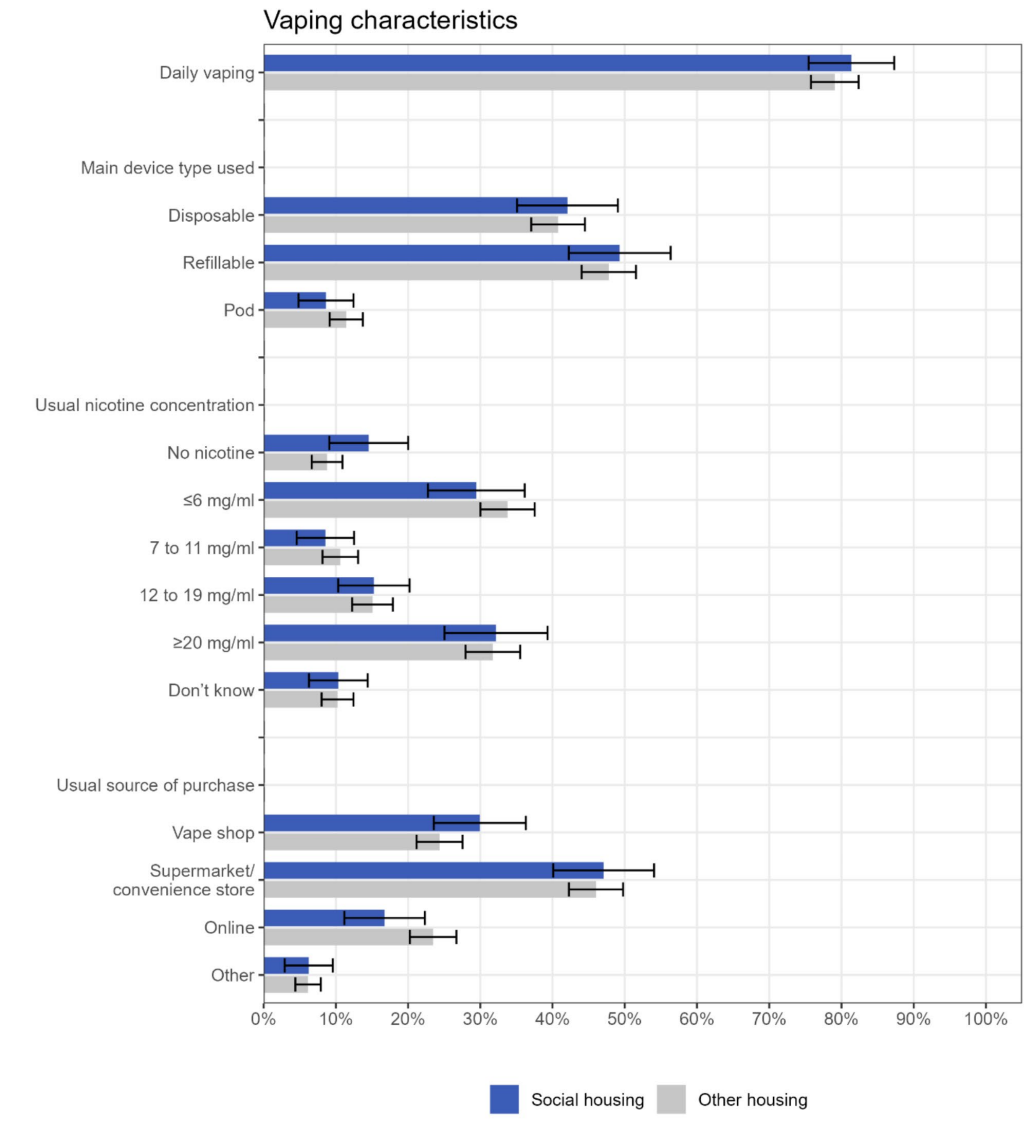
Vaping characteristics by housing tenure, among current vapers (*n* = 1,150). Data are provided in tabular form in Table S2. Unadjusted and adjusted odds ratios are provided in Table 3

Among current vapers, there were no notable differences by housing tenure (social housing vs. other housing types) in the prevalence of daily vaping, the main device type used, usual nicotine concentration, or usual source of purchase (Fig. 2; Table 3, Table S2). Likewise, there were no notable differences between those living in social housing and home owners, nor between those living in private rented accommodation and home owners (Table S3).

**Table 3:** Unadjusted and adjusted associations of housing tenure with vaping characteristics, among current vapers (*n* = 1,150)

	Vaping characteristics (social housing vs. other housing types [ref])	
	Unadjusted OR [95% CI]	Adjusted OR [95% CI]^1^
Daily vaping	1.16 [0.75–1.79]	1.17 [0.73–1.87]
Main device type used		
Disposable	1.05 [0.76–1.46]	1.09 [0.75–1.57]
Refillable	1.06 [0.77–1.46]	1.05 [0.73–1.51]
Pod	0.73 [0.43–1.24]	0.73 [0.42–1.26]
Usual nicotine concentration	0.91 [0.66–1.27]	0.90 [0.63–1.28]
Usual source of purchase		
Vape shop	1.33 [0.94–1.88]	1.25 [0.86–1.81]
Supermarket/convenience store	1.04 [0.76–1.43]	1.10 [0.78–1.55]
Online	0.66 [0.42–1.01]	0.64 [0.40–1.01]
Other	1.01 [0.53–1.93]	1.07 [0.57–2.02]

CI, confidence interval. OR, odds ratio

^1^ Adjusted for age, gender, occupational social grade, country, and smoking status. There were some missing data on each outcome (daily vaping *n* = 184, main device type *n* = 26, nicotine concentration *n* = 184, source of purchase *n* = 21). There were also some missing data on gender (*n* = 29), so the sample sizes for the adjusted models were slightly smaller.

### Use of vaping products as a smoking cessation aid

Among past-year smokers who tried to quit smoking (*n* = 1,281), just over a third reported using vaping products in their most recent past-year quit attempt, with similar rates among those living in social housing and other housing (36.0% [30.1–41.9%] vs. 36.6% [33.0-40.1%], respectively; OR = 0.98, 95%CI 0.72–1.32; OR_adj_=0.93, 95%CI 0.67–1.29). There were no notable differences between those living in social housing and home owners (OR_adj_=0.91, 95%CI 0.64–1.28), nor between those living in private rented accommodation and home owners (OR_adj_=0.94, 95%CI 0.66–1.32).

### Harm perceptions of vaping products

**Fig. 3:**
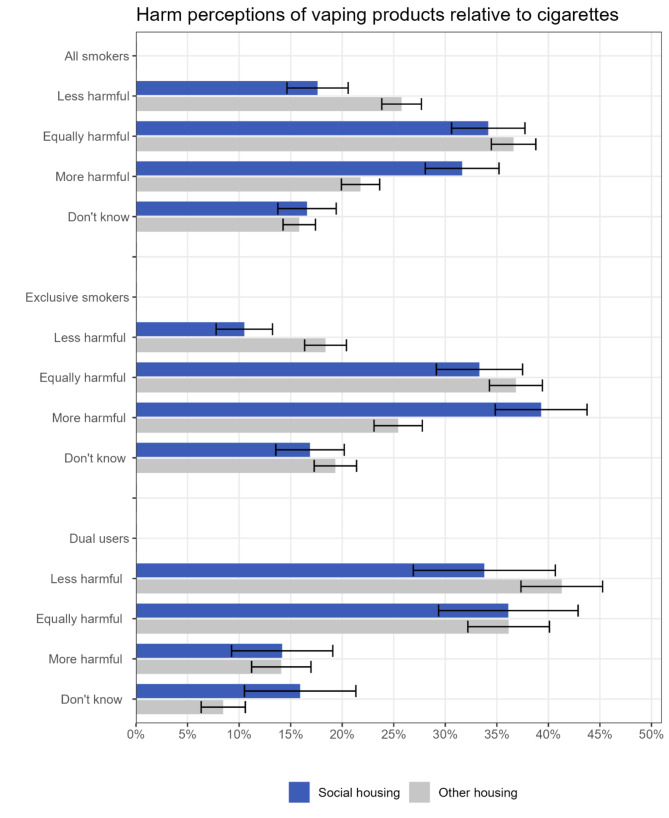
Harm perceptions of vaping products relative cigarettes by housing tenure, among current smokers (*n* = 3,390). Data are provided in tabular form in Table S4. Unadjusted and adjusted odds ratios are provided in Table 4

Among current smokers, there was a high prevalence of the belief that vaping products are more harmful than cigarettes, and the prevalence was even higher for those living in social housing than those living in other housing (Fig. 3; Table 4, Table S4). Just 17.6% of smokers living in social housing thought vaping products were less harmful than cigarettes and 65.8% thought they were equally or more harmful. For comparison, these figures were 25.8% and 58.4% respectively among smokers living in other housing. After adjustment for covariates, smokers living in social housing had lower odds of thinking vaping products were less harmful than cigarettes (OR_adj_=0.69, 95%CI 0.54–0.88) and higher odds of thinking they were more harmful (OR_adj_=1.61, 95%CI 1.30–1.99). Comparisons between smokers living in social housing and those who owned their own homes showed a similar pattern, but harm perceptions were more similar between those living in private rented accommodation and home owners (Table S5).

Perceptions were particularly inaccurate among exclusive smokers, with just 10.5% of those living in social housing thinking vaping products were less harmful than cigarettes and 72.6% thinking they were equally or more harmful. However, even among smokers who vaped (dual users), only a third (33.8%) of those living in social housing thought vaping products were less harmful. There was much more uncertainty about the relative harms among dual users living in social housing compared with dual users living in other housing types (15.9% said they did not know vs. 8.5%, respectively; OR_adj_=1.87, 95%CI 1.13–3.11).

**Table 4:** Unadjusted and adjusted associations of housing tenure with harm perceptions of vaping products relative to cigarettes, among current smokers

	Harm perceptions of e-cigarettes vs. cigarettes (social housing vs. other housing types [ref])	
	Unadjusted OR [95% CI]	Adjusted OR [95% CI]^1^
**All smokers (** *n* ** = 3,390)**		
Less harmful	0.62 [0.49–0.77]	0.69 [0.54–0.88]
Equally harmful	0.90 [0.75–1.08]	0.86 [0.71–1.05]
More harmful	1.66 [1.36–2.03]	1.61 [1.30–1.99]
Don’t know	1.06 [0.84–1.34]	0.99 [0.77–1.27]
**Exclusive smokers (*n*** **= 2**,**387)**		
Less harmful	0.52 [0.38–0.72]	0.60 [0.43–0.84]
Equally harmful	0.86 [0.69–1.06]	0.86 [0.69–1.09]
More harmful	1.90 [1.52–2.37]	1.77 [1.39–2.24]
Don’t know	0.85 [0.65–1.11]	0.81 [0.61–1.07]
**Dual users (*n*** **= 1**,**003)**		
Less harmful	0.73 [0.51–1.03]	0.78 [0.53–1.14]
Equally harmful	1.00 [0.71–1.40]	0.89 [0.61–1.30]
More harmful	1.01 [0.63–1.61]	1.15 [0.70–1.90]
Don’t know	2.05 [1.26–3.33]	1.87 [1.13–3.11]

CI, confidence interval. OR, odds ratio

^1^ Adjusted for age, gender, occupational social grade, country, and vaping status.

There were some missing data on covariates (all smokers: age *n* = 1, gender *n* = 74; exclusive smokers: age *n* = 1, gender *n* = 42; dual users: gender *n* = 32), so sample sizes for adjusted models were slightly smaller.

## Discussion

Adults living in social housing in Great Britain were more likely to vape than those living in other housing types, particularly compared with home owners. There were no notable differences by housing tenure in vaping frequency, the main device type used, usual nicotine concentration, usual source of purchase, or use as a smoking cessation aid. However, harm perceptions of vaping products relative to cigarettes were more inaccurate among current smokers living in social housing.

The prevalence of vaping was twice as high among adults living in social housing compared with those in other housing types. This was partly explained by differences in smoking status and sociodemographic characteristics. Consistent with previous studies, we found participants living in social housing were more likely to smoke [10, 17], which is linked to higher rates of vaping [13]. They were also younger, on average, than those living in other housing types. There has been a substantial rise in vaping among younger adults in Great Britain since disposable vapes became popular in 2021 [25–27], so we might expect rates of vaping to be higher among social housing residents on account of their younger age. Accordingly, the association between housing tenure and vaping attenuated after adjustment for smoking status, age, and other sociodemographic characteristics, but the adjusted odds of vaping remained 33% higher among adults living in social housing. It is possible this remaining association may be driven by network effects [28]: people living in social housing tend to live in close proximity to one another, so may be more likely to vape (independent of their own sociodemographic characteristics and smoking status) as a result of greater exposure to other people vaping. There may also be cultural reasons why disadvantaged people might want to vape (e.g., overcoming normative barriers to smoking cessation or reflecting values associated with family responsibility) [29–31]. 

Despite adults living in social housing being more likely to vape overall, vaping prevalence among those who currently smoked was similar to smokers living in other housing types. This suggests there is still substantial scope for the Swap To Stop scheme to encourage smokers living in social housing (as well as those living in other housing types – particularly private rented accommodation) to switch to vaping. However, this should be done in a careful, targeted way to avoid promoting uptake further among never smokers [25, 32]. To inform the approach, it would be useful to have more qualitative research to gain insight into the lived experience of smokers living in social housing.

Vaping characteristics were very similar across current vapers living in social housing and those living in other housing types. The majority reported vaping daily and mainly using refillable or disposable devices. The most common source of purchase was supermarkets and convenience stores, which have become more popular for buying vaping products than vape shops since the rise in popularity of disposable vapes [33]. A substantial minority reported using no or low levels of nicotine (≤ 6 mg/ml). The use of vaping products as a smoking cessation aid was also similar across housing tenures, with around one in three quit attempts involving the use of vaping products. This suggests vaping products are being used in a similar way among those who vape across housing tenures and no targeted action is needed to address inequalities in how people are vaping or the products they are using.

Harm perceptions of vaping products compared with cigarettes were generally very inaccurate but particularly so among smokers living in social housing. Despite substantial evidence indicating vaping products expose users to less harm than cigarette smoke [13], fewer than one in five smokers living in social housing thought they were less harmful than cigarettes. Two in three thought they were equally or more harmful. While exclusive smokers were more likely to believe that the harms of vaping are similar to or exceed the harms of smoking, there were also misperceptions and substantial uncertainty about the risks among dual users – particularly among those living in social housing. Addressing these misperceptions is likely to be important if the national Swap To Stop scheme is going to be successful in encouraging smokers (both in social housing and in other housing types) to switch to vaping. If exclusive smokers think vaping products are equally or more harmful than cigarettes (as the majority currently do), they may be unwilling to try them or see little benefit in switching even if offered a free starter pack, leaving them using a more harmful product [13]. Similarly, if dual users perceive the risks to be similar, then they may continue both behaviours rather than switching completely from smoking to vaping. The UK government recently committed to increased investment in national anti-smoking mass media campaigns [34], which offer a potential opportunity to correct misperceptions about vaping on a national level. This could be supplemented with local, targeted messaging aimed at people living in social housing.

Key strengths of this study were the nationally representative sample and breadth of data on vaping. There were also limitations. These data are focussed on Great Britain, which has a relatively unique tobacco control climate and approach to vaping products, and a social housing structure that may differ from other countries. Further research is needed to assess the extent to which the results generalise internationally. The items assessing vaping characteristics were only included in certain waves, reducing the sample size for these analyses. Vapers were only asked about the *main* type of device they used, which may underestimate use of specific device types if some people use multiple types. Similarly, they were asked about their usual source of purchase, which may underestimate the use of other types of retailers. However, while these issues may affect absolute estimates of prevalence, we would not expect it to affect housing tenures differently, so odds ratios should not be materially affected. Another limitation was that only current smokers were asked about harm perceptions of e-cigarettes, so we were unable to explore differences among non-smokers or those who have recently quit. In addition, our data do not offer any insight into the reasons harm perceptions differed by housing tenure. It is possible that people perceive ‘harm’ in different ways, for example in terms of direct risks to health at similar levels of consumption, concerns about different addictive potentials, or concerns that vaping might encourage children to start using nicotine. There is a need for qualitative data to better understand any specific concerns or misperceptions held by smokers in social housing.

## Conclusions

In Great Britain, adults who live in social housing are more likely to vape than those who live in other housing types (particularly home owners), even after accounting for their younger age and higher smoking rates. However, harm perceptions of vaping products relative to cigarettes are particularly negative among smokers living in social housing. Interventions addressing misperceptions of vaping could help to encourage people living in social housing to switch from smoking to vaping and reduce smoking-related health inequalities.

## Electronic supplementary material

Below is the link to the electronic supplementary material.

Supplementary Material 1

## Data Availability

The datasets analysed during the current study are available from the corresponding author on reasonable request.
