# Supplementary material for: Patterns and perceptions of vaping among adults living in social housing: a representative survey in Great Britain, 2023
Source: BMC Public Health. 2024 Sep 20;24:2572. doi: 10.1186/s12889-024-20043-5 (PMC11414268; doi:10.1186/s12889-024-20043-5)
Supplement: Supplementary file 1 — Supplementary Material 1 [file 12889_2024_20043_MOESM1_ESM.pdf]

Table S1. Current vaping prevalence by housing tenure (*n*=23,245)

	Current vaping, %[1] [95% CI]	
	**Social housing (*n*[2]=3,161)**	**Other housing (*n*[2]=20,084)**
All adults	19.4 [17.7–21.0]	10.4 [9.9–10.9]
Age (years)		
16-24	24.5 [20.0–29.1]	23.8 [21.5–26.1]
25-34	22.6 [18.3–26.8]	16.5 [14.8–18.1]
35-44	25.3 [20.7–30.0]	11.9 [10.5–13.3]
45-54	19.9 [15.6–24.1]	8.9 [7.8–10.0]
55-64	18.0 [14.2–21.9]	6.6 [5.7–7.5]
≥65	6.6 [4.6–8.5]	2.4 [1.9–2.8]
Gender		
Men	20.3 [17.7–22.9]	11.1 [10.4–11.9]
Women	18.3 [16.2–20.5]	9.5 [8.8–10.2]
Occupational social grade		
ABC1 (more advantaged)	16.1 [13.9–18.4]	8.9 [8.4–9.5]
C2DE (less advantaged)	20.6 [18.5–22.8]	12.6 [11.6–13.7]
Nation		
England	19.9 [18.1–21.8]	10.9 [10.3–11.5]
Wales	19.0 [13.1–24.9]	6.6 [5.4–7.8]
Scotland	14.1 [11.0–17.2]	7.5 [6.6–8.5]
Smoking status		
Never smoker	5.8 [4.2–7.3]	2.4 [2.0–2.7]
Ex-smoker	30.6 [26.9–34.3]	17.0 [15.7–18.2]
Current smoker	30.5 [26.9–34.1]	32.2 [30.1–34.3]

CI, confidence interval.

[1] Column percentages.

[2] Unweighted sample size.

Table S2. Vaping characteristics by housing tenure, among current vapers (n=1,150)

	%[1] [95% CI]	
	**Social housing (n^2=261)**	**Other housing (n^2=889)**
Daily vaping	81.4 [75.5–87.3]	79.1 [75.8–82.4]
Main device type used		
Disposable	42.1 [35.1–49.0]	40.8 [37.0–44.5]
Refillable	49.3 [42.2–56.3]	47.8 [44.0–51.5]
Pod	8.6 [4.8–12.4]	11.4 [9.1–13.7]
Usual nicotine concentration		
No nicotine	14.6 [9.1–20.0]	8.8 [6.7–10.9]
≤6 mg/ml	29.4 [22.7–36.1]	33.8 [30.0–37.5]
7 to 11 mg/ml	8.6 [4.6–12.5]	10.6 [8.2–13.1]
12 to 19 mg/ml	15.3 [10.3–20.2]	15.1 [12.3–17.9]
≥20 mg/ml	32.2 [25.0–39.3]	31.7 [28.0–35.5]
Don't know	10.3 [6.3–14.4]	10.2 [8.0–12.4]
Usual source of purchase		
Vape shop	29.9 [23.6–36.3]	24.4 [21.2–27.5]
Supermarket/convenience store	47.1 [40.1–54.1]	46.0 [42.3–49.8]
Online	16.7 [11.2–22.3]	23.5 [20.3–26.7]
Other	6.2 [2.9–9.6]	6.2 [4.4–7.9]

CI, confidence interval.
[1] Column percentages. There were some missing data (n=184 daily vaping, n=26 main device type used, n=23 usual nicotine concentration, n=21 usual source of purchase); valid percentages are shown.
[2] Unweighted sample size.
Data on vaping characteristics were only collected in certain waves (January, April, June, July, and October); vapers surveyed in other months were not included in these analyses.

Table S3. Unadjusted and adjusted associations of housing tenure (social housing and private rented vs. home owner) with vaping characteristics, among current vapers (n=1,127)

	**Vaping characteristics**			
	**Social housing vs. home owner**		**Private rented vs. home owner**	
	**OR [95% CI]**	**OR_{adj} [95% CI]**	**OR [95% CI]**	**OR_{adj} [95% CI]**
Daily vaping	1.10 [0.69–1.74]	1.24 [0.76–2.02]	0.90 [0.59–1.39]	1.22 [0.76–1.96]
Main device type used				
Disposable	1.23 [0.87–1.73]	1.11 [0.76–1.63]	1.59 [1.13–2.22]	1.08 [0.73–1.60]
Refillable	0.99 [0.71–1.39]	1.13 [0.78–1.65]	0.81 [0.58–1.13]	1.20 [0.81–1.77]
Pod	0.61 [0.35–1.05]	0.61 [0.35–1.09]	0.52 [0.30–0.92]	0.55 [0.30–0.99]
Usual nicotine concentration	0.92 [0.65–1.29]	0.81 [0.56–1.16]	1.04 [0.76–1.42]	0.79 [0.56–1.12]
Usual source of purchase				
Vape shop	1.23 [0.86–1.78]	1.17 [0.79–1.72]	0.85 [0.58–1.26]	0.88 [0.59–1.33]
Supermarket/convenience store	1.17 [0.84–1.63]	1.16 [0.81–1.67]	1.33 [0.96–1.86]	1.13 [0.79–1.61]
Online	0.62 [0.39–0.97]	0.65 [0.41–1.04]	0.86 [0.58–1.28]	1.06 [0.69–1.64]
Other	0.96 [0.49–1.87]	1.00 [0.52–1.93]	0.74 [0.36–1.55]	0.77 [0.37–1.60]

CI, confidence interval. OR, odds ratio. OR_{adj}, odds ratio adjusted for age, gender, occupational social grade, country, and smoking status.

There were some missing data on each outcome (daily vaping *n*=180, main device type *n*=26, nicotine concentration *n*=144, source of purchase *n*=20). There were also some missing data on gender (*n*=28), so the sample sizes for the adjusted models were slightly smaller.

Table S4. Unadjusted and adjusted associations of housing tenure with harm perceptions of vaping products relative to cigarettes, among current smokers (*n*=3,390)

	%[1] [95% CI]	
	**Social housing**	**Other housing**
**All smokers**		
Unweighted *N*	867	2,523
Less harmful	17.6 [14.6–20.6]	25.8 [23.8–27.7]
Equally harmful	34.2 [30.6–37.7]	36.6 [34.5–38.8]
More harmful	31.6 [28.1–35.2]	21.8 [19.9–23.6]
Don't know	16.6 [13.8–19.4]	15.8 [14.3–17.4]
**Exclusive smokers**		
Unweighted *N*	625	1,762
Less harmful	10.5 [7.8–13.2]	18.4 [16.4–20.4]
Equally harmful	33.3 [29.1–37.5]	36.8 [34.3–39.4]
More harmful	39.3 [34.8–43.7]	25.4 [23.1–27.8]
Don't know	16.9 [13.6–20.2]	19.3 [17.3–21.4]
**Dual users**		
Unweighted *N*	242	761
Less harmful	33.8 [26.9–40.7]	41.3 [37.3–45.3]
Equally harmful	36.1 [29.3–42.9]	36.1 [32.2–40.1]
More harmful	14.2 [9.3–19.1]	14.1 [11.2–17.0]
Don't know	15.9 [10.5–21.3]	8.5 [6.3–10.6]

CI, confidence interval.

[1] Column percentages.

Table S5. Unadjusted and adjusted associations of housing tenure with harm perceptions of vaping products relative to cigarettes, among current smokers

	Harm perceptions of e-cigarettes vs. cigarettes			
	**Social housing vs. home owner**		**Private rented vs. home owner**	
	**OR [95% CI]**	**OR_{adj} [95% CI]**	**OR [95% CI]**	**OR_{adj} [95% CI]**
**All smokers (*n*=3,294)**				
Less harmful	0.58 [0.46–0.74]	0.65 [0.50–0.84]	0.87 [0.70–1.08]	0.87 [0.68–1.12]
Equally harmful	0.88 [0.73–1.07]	0.86 [0.69–1.06]	0.95 [0.78–1.17]	0.97 [0.78–1.20]
More harmful	1.87 [1.51–2.32]	1.69 [1.35–2.13]	1.41 [1.12–1.77]	1.19 [0.92–1.53]
Don't know	1.02 [0.79–1.30]	1.02 [0.78–1.32]	0.84 [0.65–1.10]	1.03 [0.77–1.38]

CI, confidence interval. OR, odds ratio. OR_{adj}, odds ratio adjusted for age, gender, occupational social grade, country, and vaping status.

There were some missing data on covariates (age *n*=1, gender *n*=68), so sample sizes for adjusted models were slightly smaller.
